# A phylogenetic analysis of the biting midges belonging to *Culicoides* Latreille (Diptera: Ceratopogonidae) subgenus *Avaritia* using molecular data

**DOI:** 10.1186/s13071-020-04111-4

**Published:** 2020-05-12

**Authors:** Bruno Mathieu, Claire Garros, Thomas Balenghien, Ermanno Candolfi, Jean-Claude Delécolle, Catherine Cêtre-Sossah

**Affiliations:** 1 IPPTS, Université de Strasbourg, DIHP UR 7292, 67000 Strasbourg, France; 2 ASTRE, Univ Montpellier, Cirad, INRA, Montpellier, France; 3 Cirad, UMR ASTRE, F-34398 Montpellier, France; 4 CIRAD, UMR ASTRE, Rabat, Morocco; 5 Unité Microbiologie, Immunologie et Maladies Contagieuses, IAV Hassan II, Rabat, Morocco; 6 CIRAD, UMR ASTRE, Sainte Clotilde, La Réunion, France

**Keywords:** *Culicoides*, phylogeny, taxonomy, species group, systematics

## Abstract

**Background:**

Within the genus *Culicoides* (Diptera: Ceratopogonidae), the subgenus *Avaritia* is of particular interest as it contains a significant number of economically important vector species. Disagreements about the systematic classification of species within this subgenus have resulted in a taxonomic imbroglio.

**Methods:**

A molecular phylogeny of the subgenus *Avaritia* was conducted to test the existing systematic classification, which is based on phenetic assessment of morphological characters. Three nuclear ribosomal markers, internal transcribed spacer 1 and 2 (ITS1, ITS2), *5.8S*, and three mitochondrial markers, cytochrome *c* oxidase subunit 1 and 2, and cytochrome b (*cox*1, *cox*2 and *cytb*), were obtained for 37 species of the subgenus *Avaritia* from all six biogeographical regions. Phylogenetic reconstructions using these genes independently and in combination were implemented using Bayesian inference analysis and maximum likelihood methods.

**Results:**

Phylogenetic reconstructions gave strong support to several monophyletic groups within the subgenus *Avaritia.* Both *C. actoni* and *C. pusillus* formed a single clade with *C.* *grahamii* so their respective groups, the Actoni and Pusillus groups, have been merged with the Grahamii group. Some support was provided for the Boophagus and Jacobsoni groups. A group of species currently placed into the Orientalis group clustered in a clade with poor support. The Obsoletus group was defined as a sister clade to all other *Avaritia* groups. The clade including the Imicola group was well supported based on phylogenetic criteria.

**Conclusions:**

This phylogenetic study combining five distinct molecular markers has provided meaningful insights into the systematic relationships of *Culicoides* (*Avaritia*) and highlighted future directions to continue the study of this subgenus. While the *cox*2 marker appeared to be useful to investigate closely related species, the *5.8S* marker was highly conserved and uninformative. Further investigations including species absent from this work are needed to confirm the proposed systematic scheme. However, this systematic scheme can now serve as a foundation to investigate cryptic species affiliation within the subgenus. We advocate that future studies employ a combination of morphological and molecular analyses.
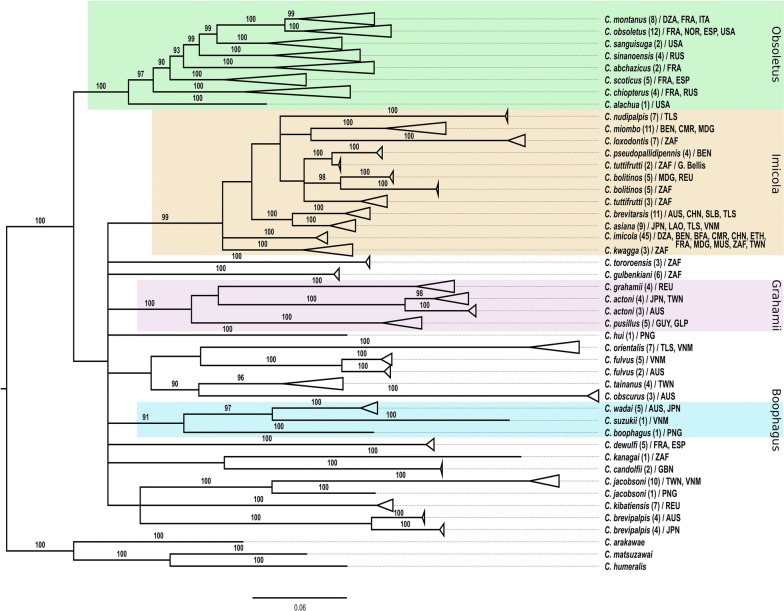

## Background

The genus *Culicoides* Latreille (Diptera: Ceratopogonidae) is one of the most diverse groups of ceratopogonids, with over 1400 described species worldwide [1]. The subgeneric classification of the genus has traditionally relied on morphological characters such as variations in wing patterns and male genitalia [2]. The current subgeneric classification consists of 31 subgenera containing 63% of extant species, 38 unplaced groups of species made of 24% of extant species and a further 13% of extant species that have not been placed into any of these groups [1, 2].

Attempts to subdivide this large genus into subgenera have been partially successful. Adult morphological characters have most often been used to define these groupings although Glukhova [3] also relied on larval characters. Root & Hoffmann [4] and Edwards et al. [5] first proposed division of the genus into two series based on the male genitalia and other external features. Khalaf [6] subsequently attempted a classification based on features of male genitalia and female spermathecae but these two classification schemes are divergent in their grouping delimitation and species composition.

Starting in the 1950’s, some authors felt uncomfortable assigning species to untested subgenera and preferred to gather species in groups, many of which were not assigned to a subgenus [7, 8]. These contributions to the subgeneric classification were often based on regional assessments with limited attempts to rationalize groupings leading to numerous different species groups some of which were likely synonymous [9–11]. This approach was first used by Root & Hoffman [4] who separated the North American species into two “series”. Khamala & Kettle [8] preferred to group the 61 East African species into 16 species groups whereas Wirth & Blanton [9] grouped 88 species from Panama into 21 species groups and Campbell & Pelham-Clinton [10] placed 41 British species in 11 species groups. Partly due to the difficulty to collaborate in those days in terms of accessibility to material and communication, the authors classified the fauna based on their own geographical studies rather than placing them in context with an overarching assessment. Therefore, without any accordance on species groupings and their composition, duplication of species groupings and names emerged [2].

Confounding this main issue is the proliferation in use of the terms species group and species complex [2, 11]. The difficulty is that the International Code of Zoological Nomenclature (ICZN) does not recognize these levels as having a formal status even though their use is prevalent within *Culicoides* literature [2]. The knowledge of the phylogenetic relationships between species, complexes and groups within subgenera is therefore very incomplete and has not yet been evaluated worldwide using a multi-molecular markers approach. Recent molecular phylogenetic studies suggest that at least some of the current subgenera are polyphyletic [12–15], prompting Borkent to consider the current classification as in “chaos” [1].

Shortly after the classification attempt by Khalaf [6], Fox [16] erected the subgenus *Avaritia* to encompass Khalaf’s Chiopterus species group. This subgenus was subsequently validated [9, 17] with the definition by Wirth & Hubert [9] being the most widely accepted description of the subgenus. The definition of Wirth & Hubert was, however, not followed by Yu et al. [18] who adopted a very different diagnosis resulting in the inclusion of a large number of Chinese species that Wirth & Hubert had affiliated to different groups or subgenera. Indeed, Dyce et al. [19] removed the species represented in Australasia back into the groupings defined by Wirth & Hubert [17] and added the Jacobsoni complex to the growing number of natural groupings within subgenus *Avaritia* [3, 17, 20].

The subgenus *Avaritia* includes species known to be vectors of viruses of ruminants and equids distributed worldwide causing economically important diseases [21]. The first attempt at a worldwide systematic scheme within subgenus *Avaritia* was by Meiswinkel and colleagues [11, 22] who listed all species into the six species “complexes” known at the time: the Andicola, Grahamii (=Actoni *sensu* Dyce et al. [19]), Imicola, Obsoletus, Pusillus and Orientalis complexes. A tentative Pseudopallidipennis “subgroup” of the Imicola group as well as two further complexes (Gulbenkiani and Suzukii) were proposed. Indeed, each of these groupings had been subject to revision from several authors throughout the years. We propose hereafter a clarification for each group.

Khamala & Kettle [8] used adult features to delimit East Afrotropical species groups and grouped seven species (*C.* *imicola* Kieffer (syn. *C.* *pallidipennis*), *C.* *grahamii* Austen, *C.* *kibatiensis* Goetghebuer, *C.* *trifasciellus* Goetghebuer, *C.* *tororoensis* Khamala & Kettle, *C.* *kanagai* Khamala & Kettle and *C.* *glabripennis* Goetghebuer (syn. *C.* *spinifer*)) into the Pallidipennis group, which they thought was related to the Obsoletus group of Root & Hoffman [4] and Campbell & Pelham-Clinton [10] and the subgenus *Avaritia* described by Wirth & Blanton [23]. Glick [7] changed the name of the group to the Imicola group after the synonymisation of *C.* *pallidipennis* with *C.* *imicola.* Due to the economic importance of several species of the group, it has received great attention. *Culicoides imicola* and related species were placed into the Orientalis group by Wirth & Hubert [17], whereas other authors have clearly distinguished the Imicola and the Orientalis groups [11]. Glick [7] did not refer to the subgenus *Avaritia* and placed all the Ethiopian species into one Imicola group, including species which are currently not considered closely related to *C.* *imicola*, e.g. *C.* *kanagai.* More recently Chang et al. [24] provided a checklist of the Chinese species belonging to the subgenus *Avaritia* but followed the systematic system of Yu et al. [18] which included species that do not comply with the more widely accepted definitions of the subgenus and without any mention of groups within the subgenus. Meiswinkel [11] redefined the Imicola and the Orientalis groups based on adult features assigning 11 species to the Orientalis group and 12 species (including 3 undescribed species) to the Imicola group. In addition, Meiswinkel’s Pseudopallidipennis subgroup has been considered as a full species complex [25] though needing possible amendment on its delimitation [11].

The Andicola group was originally and adequately defined by Wirth & Lee [26] for three new species collected from high altitude sites in the Colombian Andes. More recently, two new species, also collected at high altitudes, were assigned to the Andicola group [27, 28] which now includes five Neotropical species (*C.* *andicola* Wirth & Lee, *C.* *hermani* Spinelli & Borkent, *C.* *puracensis* Wirth & Lee, *C.* *orjuelai* Wirth & Lee, and *C.* *suarez* Rodriguez & Wirth).

Wirth & Hubert [17] first defined the Actoni species group to accomodate the Oriental, Afrotropical and Australasian regions encompassing *C.* *actoni*, *C.* *dasyops* Clastrier and *C.* *minimus* Wirth & Hubert, and transferred *C.* *grahamii* from the Imicola group. Their diagnosis, species with pubescent eyes and the second radial cell being completely dark, overlapped with that of the Pusillus Group creating some confusion about the boundaries of these groups. Meiswinkel et al. [22] subsequently renamed this group after *C.* *grahamii* following the convention of naming a group after the oldest known member.

The Pusillus species complex of Wirth et al. [29] includes three species (*C.* *pusillus* Lutz, *C.* *pusilloides* Wirth & Blanton, and *C.* *impusilloides* Spinelli & Wirth) but so far lacks a precise definition.

Meiswinkel et al. [22] defined two additional species groups, the Gulbenkiani group for *C.* *brevipalpis* Delfinado, *C.* *gulbenkiani* Caeiro and *C.* *tororoensis*, and the Suzukii group including *Culidoides* *wadai* Kitaoka. The latter was referred to as the Boophagus group by Dyce et al. [19] as *C.* *boophagus* is the oldest described member. More recently, Bellis et al. [30] supported the monophyly of the Imicola complex and excluded *C.* *brevipalpis* from that species group based on morphological criteria and molecular analysis. In 2001, Dyce [31] was the first to mention a Jacobsoni group but neither this article nor Dyce’s pictorial atlas published in 2007 [19] provide a diagnosis of this group.

Campbell & Pelham-Clinton [10] revised the taxonomic scheme of the British *Culicoides* fauna and proposed the Obsoletus group comprising the Chiopterus group of Khalaf [6] but excluding *C.* *austeni* Carter, Ingram & Macfie and including *C.* *sinanoensis* Tokunaga, *C.* *jacobsoni* Macfie (syn. *C.* *kitaokai* Tokunaga) and *C.* *brevitarsis* Kieffer (syn. *C.* *robertsi* Lee & Reye). The Palaearctic species of this group have recently received increasing attention due to the association of several species with outbreaks of bluetongue and Schmallenberg diseases [32, 33]. Due to similarities in wing patterns with *C.* *obsoletus* (Meigen), *C.* *dewulfi* Goetghebuer was considered part of the Obsoletus group [10, 34].

The lack of agreement of morphological characters that define subgenus *Avaritia* and its component species groups is fundamental to the confusion around the status and composition of species groups within the subgenus. An independent means of testing the limits of these groups would lend confidence to their status and help clarify those morphological characters which have phylogenetic importance. Molecular phylogenetic studies have been used successfully to test systematic proposals of many types of organisms [35, 36] that are based on morphological evidence and could prove useful in clarifying the classification of subgenus *Avaritia.*

A range of markers have been used to test the phylogeny of *Culicoides* (*Avaritia*) with varying success. One study combining data from *cox*1 and *cox*2 [37] did not support the monophyly of the subgenus, casting doubt over the usefulness of mitochondrial markers for higher-level phylogenetic studies. Other studies have, however, successfully used *cox*1 to investigate relationships at a lower level for example, within the Imicola group [13, 38]. Studies using either internal transcribed spacer 1 (ITS1) [39] or 2 (ITS2) [40] alone or in combination [41] have also been used successfully to investigate species relationships within subgenus *Avaritia.* Additionally, *cox*1 has been combined with other markers in a multi-marker approach to investigate the phylogeny of *Culicoides* (*Avaritia*). While a first study based on *cox*1 and *CAD* (carbamoyl phosphate synthetase 2) supported the monophyly of the Imicola complex [30], a second has reported support for several subgeneric groupings and highlighted the presence of cryptic species [42]. Combining *cox*1, ITS1 and ITS2, provided evidence to exclude *C.* *dewulfi* from the Obsoletus group [15]. In 2018, a comprehensive phylogenetic study of the Afrotropical fauna, including several species from subgenus *Avaritia*, used two mitochondrial (*cox*1 and *16S* rDNA) and two nuclear (*CAD* and *28S* rDNA) markers to support the monophyly of the Imicola and Dasyops groups [43].

This study aims to (i) test species group delimitation within subgenus *Avaritia*; (ii) elucidate the phylogenetic relationships among the different species groups; and (iii) propose a new systematic scheme of the subgenus *Avaritia*, at a worldwide level, using six genetic markers including nuclear and mitochondrial molecular targets (ITS1, ITS2, *5.8S*, *cox*1, *cox*2, *cytb*).

## Methods

### Taxon sampling and species identification

A total of 191 specimens representing 35 species belonging to *Culicoides* (*Avaritia*) were collected from 21 countries from all six biogeographical regions of the world (Additional file 1: Table S1). The sampling effort was heavily represented by specimens from the Palaearctic and Afrotropical regions. Species identification was determined or confirmed by either the experts who kindly provided specimens or by the authors of this paper using the following morphological studies and keys [7, 8, 17, 19, 44–47]. *Culicoides* (*Meijerehelea*) *arakawae* (Arakawa), the only species of *Culicoides* with publicly available mitochondrial and rDNA sequences, was selected as the outgroup. Two additional species belonging to *Culicoides* (*Trithecoides*), i.e. *C.* (*T*.) *humeralis* Okada and *C.* (*T.*) *matsuzawai* Tokunaga, were also added as outgroups because of the availability of three of the five genetic markers used in this study.

### DNA extraction, genome amplification by PCR and sequencing

A semi-destructive method was used to extract DNA from specimens. Each specimen was soaked in a saturated phenolic solution before dissection and mounted in Canada balsam following the procedure described in [48] except for the thorax and the first five abdominal segments that were retained for DNA extraction. Total genomic DNA was extracted from the tissues digested with Proteinase K at 10%, using the DNeasy Tissue kit (Qiagen, Valencia, CA, USA) following the manufacturer’s instructions.

In addition to the barcode region cytochrome *c* oxidase subunit 1 (*cox*1), cytochrome *c* oxidase subunit 2 (*cox*2) and cytochrome b (*cytb*) mitochondrial gene fragments (mt) were amplified. One ribosomal DNA (rDNA) gene fragment was studied: the internal transcribed spacer 1 and 2 (ITS1 and ITS2) including the coding *5.8S* region. PCR reactions were performed in a total volume of 50 µl consisting of 1× PCR reaction buffer, 2.5 mM of MgCl_2_, 250 μM each of dATP, dCTP, dGTP and dTTP (Invitrogen, Carlsbad, CA, USA), 200 ng of each primer, and 2.5 U of HotStart Taq DNA polymerase (Qiagen) and 1 μl of template DNA. Primer sets used are listed in Table 1. PCR cycling conditions for mtDNA were as follows: an initial denaturation step at 95 °C for 15 min, then 5 cycles at 95 °C for 40 s, 45 °C for 40 s, 72 °C for 1 min, followed by 45 cycles at 95 °C for 40 s, 50 °C for 40 s, 72 °C for 1 min and a final extension step at 72 °C for 20 min. To amplify the ITS1-5.8S-ITS2 region, the PCR cycling conditions were as follows: an initial denaturation step at 95 °C for 15 min, then 40 cycles at 95 °C for 30 s, 54 °C for 1 min, 72 °C for 1 min and a final extension step at 72 °C for 20 min. PCR products were examined by electrophoresis in a 2% ethidium bromide agarose gel.

**Table 1:** Primers used to amplify the five molecular regions of *Culicoides* specimens

Gene	Primer name	Sequence 5′–3′	Fragment length (bp)	Reference
*cox*1	C1J1718	GGAGGATTTGGAAATTGATTAGT	523	Dallas et al. [38]
	C1N2191	CAGGTAAAATTAAAATATAAACTTCTGG		Dallas et al. [38]
*cox*2	COIIF20	ATGGCAACTTGAGGAMATAT	601	This study
	COIIR612	CGCAGATTTCTGAACATTG		This study
*cytb*	CytbF373	ATAGGAACTGCTTTTATAGG	526	This study
	CytbR944	CAATAGATATGACTAAAGCGATTACT		This study
ITS1-5.8S-ITS2	PanCulF	GTAGGTGAACCTGCGGAAGG	~785	Cêtre-Sossah et al. [67]
	28SR	ATTTGGGGGTAGTCACACAT		Gomulski et al. [40]

*Note*: The 3 regions ITS1-58S-ITS2 were amplified together

*Abbreviations*: *cox*1, cytochrome *c* oxidase subunit 1; *cox*2, cytochrome *c* oxidase 2; *cytb* for cytochrome b; ITS1-58S-ITS2, internal transcribed spacer 1-5.8S-internal transcribed spacer 2

PCR products were sequenced in both directions by Eurofins MWG Operon (Edersberg, Germany). The rDNA ITS1-5.8S-ITS2 region was sequenced after a cloning step into the PCR-Blunt vector (Zero Blunt PCR Cloning Kit, Invitrogen), using chemically competent *E. coli*. Plasmid DNA was extracted using a Plasmid DNA Preparation Kit (Nucleospin® Plasmid, Macherey Nagel, USA). *Bam* HI (Biolabs, UK) restriction endonuclease digestion and quantification of DNA plasmid copies were carried out by measuring with a spectrophotometer DNA concentration (wavelength of 280 nm) before sequencing.

### Datasets

Among the 35 species investigated in this study, the number of specimens of each species successfully sequenced for each marker is presented in Table 2.

**Table 2:** List of the 191 specimens representing 35 species of *Culicoide* (*Avaritia*) collected and the corresponding number of specimens successfully sequenced for *cox*1, *cox*2, *cytb* and ITS1-5.8S-ITS2 markers

Species	n	cox1	cox2	cytb	ITS1-5.8S-ITS2
*C. abchazicus* Dzhafarov	2	2	2	1	2
*C. actoni* Smith	5	5	0	3	3
*C. alachua* Jamnback & Wirth	1	1	0	1	1
*C. asiana* Bellis	1	1	0	1	0
*C. bolitinos* Meiswinkel	8	8	4	8	0
*C. boophagus* Macfie	1	1	1	1	1
*C. brevipalpis* Delfinado	4	4	4	1	3
*C. brevitarsis* Kieffer	5	5	5	4	3
*C. chiopterus* (Meigen)	4	4	3	0	2
*C. dentiformis* McDonald & Lu	3	0	0	0	0
*C. dewulfi* Goetghebuer	6	5	6	6	3
*C. fulvus* Sen & Das Gupta	7	7	0	7	2
*C. grahamii* Austen	4	4	0	1	2
*C. gulbenkiani* Caeiro	5	5	2	5	4
*C. imicola* Kieffer	42	38	39	34	3
*C. jacobsoni* Macfie	9	9	1	9	1
*C. kanagai* Khamala & Kettle	1	1		1	1
*C. kibatiensis* Goetghebuer	7	7	0	6	3
*C. loxodontis* Meiswinkel	4	4	4	0	1
*C. miombo* Meiswinkel	11	9	7	10	2
*C. montanus* Shakirzjanova	8	7	3	8	4
*C. nudipalpis* Delfinado	2	2		1	0
*C. obscurus* Tokunaga & Murachi	3	3	3	0	3
*C. obsoletus* (Meigen)	12	10	2	11	6
*C. orientalis* Macfie	6	6	2	1	0
*C. pseudopallidipennis* Clastrier	3	3	2	2	1
*C. pusillus* Lutz	5	5	1	2	3
*C. sanguisuga* (Coquillett)	2	2	1	2	2
*C. scoticus* Downes & Kettle	5	2	5	1	2
*C. sinanoensis* Tokunaga	4	4	0	3	2
*C. suzukii* Kitaoka *sensu* Lien	1	1	0	0	0
*C. tainanus* Kieffer	3	2	0	3	0
*C. tororoensis* Khamala & Kettle	3	3	0	0	2
*C. tuttifrutti* Meiswinkel, Cornet & Dyce	1	1	1	1	1
C. wadai *Kitaoka*	3	3	3	3	3
Total	191	174	101	137	66

*Abbreviations*: *cox*1, cytochrome *c* oxidase subunit 1; *cox*2, cytochrome *c* oxidase 2; *cytb* for cytochrome b; ITS1-58S-ITS2, internal transcribed spacer 1-5.8S-internal transcribed spacer 2; n, number of specimens

For each marker, the complete list of specimens, species names, specimen codes, accession numbers including the sequences retrieved from GenBank are provided in Additional file 1: Table S1. For only one species, *C.* *dentiformis* McDonald & Lu from Taiwan, were no sequences available for any of the 5 amplified genetic markers. *cox*1 barcodes for *C.* *candolfii* Delécolle, Paupy, Rahola & Mathieu, *C. hui* Wirth & Hubert and C. *kwagga* Meiswinkel were retrieved from GenBank and added to the dataset. As a result, the complete dataset includes 37 species. The *cox*1 dataset contained 174 specimens from 21 countries representing 34 species, while 101 *cox*2 sequences were generated for 22 species from 18 countries. For *cox*1 and *cox*2, respectively 15 and 8 sequences from GenBank were added. The *cytb* dataset included 138 sequences representing 27 species from 20 countries while the ITS1+ITS2 dataset contained 67 sequences representing 28 species from 17 countries, with an additional 9 sequences retrieved from GenBank. Sequences of *C.* *arakawae* for the five genetic markers of the study were added to the dataset.

### Alignment and phylogenetic analyses

The mtDNA sequences (*cox*1, *cox*2 and *cytb*) were originally aligned using Vector Nti v11.5.0 (Invitrogen) and checked using ClustalW [49]. Alignment of non-coding rDNA sequences (ITS1, 5.8S, ITS2) was performed with MAFFT [50] and gaps were removed using MEGA version 5 [51]. Alignments of each of the mtDNA regions were translated into peptide sequences using MEGA version 5 [51] in order to exclude putative NUMt copies.

Heterogeneity tests of nucleotide frequency among specimens were performed using DAMBE [52]. Using the same software, potential saturation of each dataset was investigated graphically by plotting the absolute number of transitions and transversions against Tamura-Nei model (referred as ML Composite TN93) distance for all pairwise comparisons of sequences. The sequences revealing a saturated phylogenetic signal were discarded from the final dataset. The remaining sequences were concatenated using Seaview [53] to be analysed as a whole fragment with a partitioning strategy as described below.

Phylogenetic reconstructions were performed for each dataset using both non-probabilistic and probabilistic approaches. The non-probabilistic approach, maximum parsimony (MP), was performed using MEGA with bootstrapping method (*n* = 200) considering all sites informative (missing data non-excluded) and with the Close-Neighbor-Interchange (CNI) search method on Random Trees. The probabilistic approaches were Maximum Likelihood (ML) and Bayesian inference (BI). To define model parameters, the best-fit model of nucleotide substitutions was calculated with JModelTest v.0.1.1 [54] using the selection of the Akaike information criterion (AIC). The general time reversible (GTR) + I + Γ model was indicated as the best-fit model for the *cox*1, *cox*2 and *cytb* considered separately. The GTR + Γ model was the best-fit one for the ITS1 and the ITS2 considered separately whereas the Hasegawa Kishino and Yano (HKY) + Γ model was indicated for the combined ITS1+ITS2 dataset. The three mtDNA genes as a combined dataset followed the GTR + Γ model.

ML phylogenetic analyses were carried out with PhyML v3.0 [55] according to the respective best-fit model. The transition/transversion ratio, the Γ parameter and if necessary, the proportion of invariant sites were estimated. The starting tree was determined by BioNJ analysis and the branch was tested by the Shimodaira-Hasegawa-like branch test.

Phylogenetic analyses were also carried out under BI using MrBayes v3.1.2 [56]. To increase the fit of evolutionary models with data, we used partitioned analyses, which allow subsets of the data to evolve under distinct models and parameters [57, 58]. Three partitioning strategies were defined *a priori*: strategy P1, corresponding to an unpartitioned analysis; strategy P2, implementing one partition for the mitochondrial genes and one partition for the nuclear markers (ITS1+ITS2); and strategy P3, which implements a partition for each genetic marker *cox*1, *cox*2, *cytb* and ITS1+ITS2. For each partitioning strategy, independent runs of 2,000,000 generations were carried out applying appropriate models of evolution to each partition. A total number of 5000 of the saved trees were discarded and the remaining 15,000 trees were used to construct the BI trees. Clade posterior probabilities (cpp) estimates were used to assess the robustness of tree nodes. The best-fit partitioning strategy was determined by comparing the Bayes factors (B_F_) estimated as twice the difference of harmonic means.

## Results

### Molecular phylogeny analysis

**Fig. 1:**
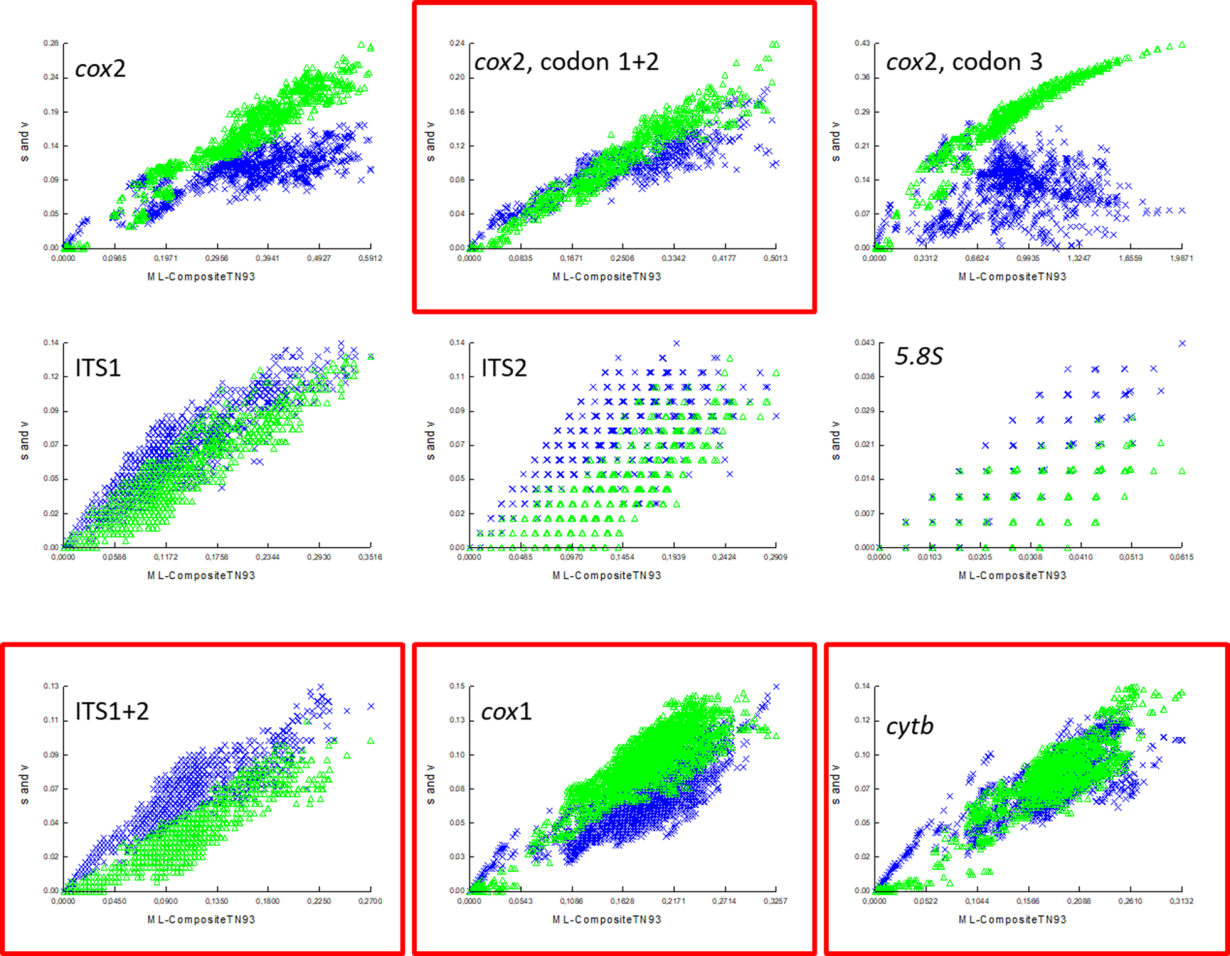
Saturation plots of the absolute numbers of transitions (blue) and transversions (green) for each genetic marker and for the combined ITS1+ITS2. The red boxes highlight the best phylogenetic signal which was then retained for analysis

Heterogeneity tests on nucleotide frequency were not significant for all the genes except for *cox*2 (*χ*^2^ = 607.43, *df* = 330, *P* < 0.001). The heterogeneity of nucleotide frequency for *cox*2 investigated by codon position showed homogeneity on codons 1+2 (*χ*^2^ = 160.65, *df* = 330, *P* = 1), and heterogeneity on codon 3 (*χ*^2^ = 1672.44, *df* = 330, *P* < 0.001) with high transition saturation rate. The *cox*2 gene was therefore analysed only on codon positions 1+2 (Fig. 1). The saturation plot for the *5.8S* gene showed transversion information saturation indicating that this region is highly conserved and uninformative; this locus was removed from the analysis. The exclusion of indels from alignments of the ITS1 and ITS2 datasets produced short fragments of 221 bp and 127 bp, respectively. The two ITS regions were analysed independently and concatenated (Fig. 1). No saturation of the phylogenetic signal was observed for the *cox*1 and *cytb* dataset.

Phylogenetic reconstructions were carried out on four datasets comprising *cox*1, *cox*2 (codons 1+2), *cytb* and ITS1+ITS2, representing 498-bp, 352-bp, 507-bp and 348-bp fragments, respectively. A combined dataset was also subjected to analysis with all these datasets giving a concatenated 1705 bp long fragment.

**Fig. 2:**
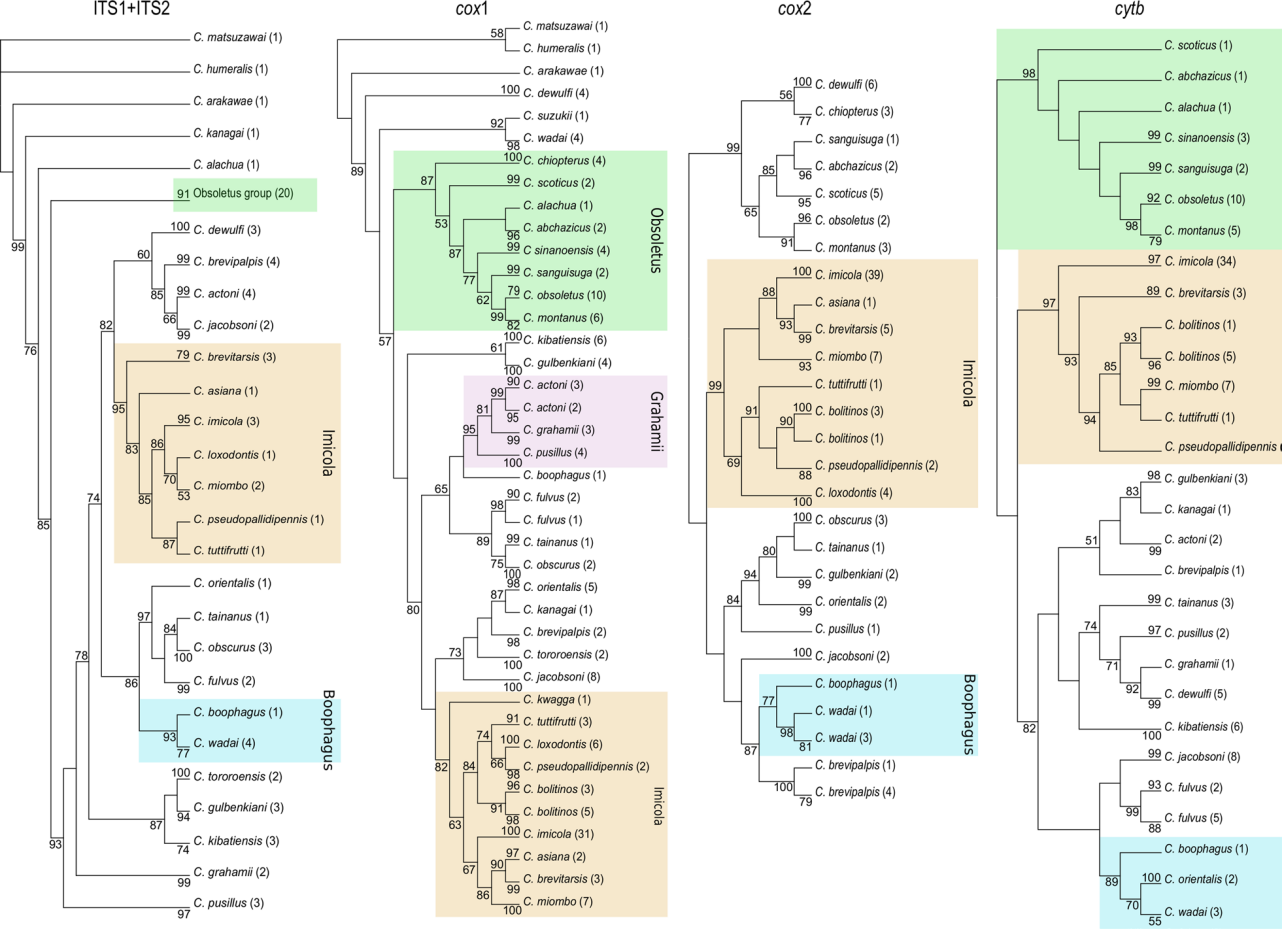
Trees obtained by independent ML reconstruction for each gene region. Data sets did not include any gaps and missing data. Orange, green, purple and blue boxes outline the four species groups, i.e. Imicola, Obsoletus, Grahamii and Boophagus, respectively, supported by a SH-like value higher than 80% in this study. SH-like percentages of 200 replicates are shown above the branches for values exceeding 50%. Trees represent ITS1+ITS2, *cox*1, *cox*2, and *cytb* marker. The clade labelled Obsoletus group in ITS1+ITS2 has been collapsed to provide a better insight of topology, and includes *C.* *abchazicus*, *C.* *chiopterus*, *C.* *montanus*, *C.* *obsoletus*, *C.* *sanguisuga*, *C.* *scoticus* and *C.* *sinanoensis*

As tree reconstructions using MP and ML resulted in similar topologies and identical species clade composition regardless of the dataset used, only results produced using ML are presented (Fig. 2). Although similarity in topologies with ML were observed, clades representing species groups were not strongly supported (bootstrap values less than 50%) in MP reconstruction, except for the *cytb* marker for which the clade Imicola group was supported by a 92% bootstrap value (data not shown).

### Maximum likelihood reconstruction

Figure 2 represents the ML reconstructions for the four datasets analysed. Three main clades were identified representing species respectively placed into the Obsoletus, Imicola and Grahamii groups. The analysis of the ITS1+ITS2 region showed a well-supported basal topology and these three groups were well supported with SH-Like values above 91%. Intra-group nodes were quite well supported for the Imicola and Grahamii groups but not for the Obsoletus group (not shown as this latter clade is collapsed to provide a better insight of topology).

The Imicola group clade was well supported in each analysis although two clades were consistently observed for *C.* *bolitinos* Meiswinkel (Fig. 2). The Obsoletus group clade was supported by *cox*1, *cytb* and ITS1+ITS2 but the *cox*2 analysis differed as it included *C.* *dewulfi* in this group (Fig. 2). The basal part of the tree topologies of the *cox*1 dataset showed lower support than the other datasets.

### Bayesian inference on combined dataset

**Fig. 3:**
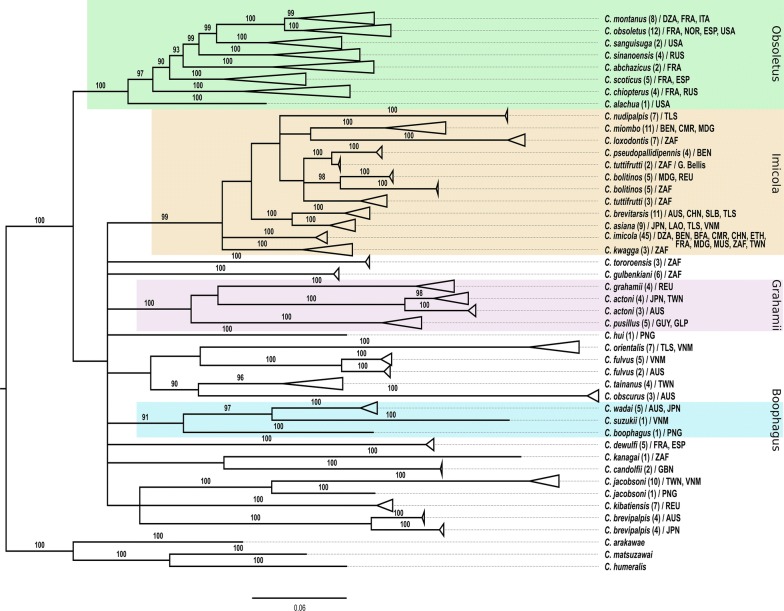
Bayesian tree resulting from the phylogenetic analysis of the combined dataset according the best-fit partitioning strategy. Robustness of nodes was indicated by the clade posterior probability values (cpp in %). Boxes are drawn to gather species belonging to a group supported by a cpp-value higher than 90%. Green, orange, purple and blue boxes outlining the four supported groups in this study, i.e. Obsoletus, Imicola, Grahamii and Boophagus, respectively. For each species, the number of specimens used in the analysis is provided in parentheses and their country origin is referred using the ISO-3 country code

Comparison of the Bayes factor obtained from the three distinct partitioning strategies on the combined data run independently favours the strategy P2. No topology incongruence was detected between well-supported nodes on separate ML analysis and the combined analyses for the three BI strategies. Moreover, the combined analysis showed a well-supported topology of both deep and terminal nodes; the strategy P2 was therefore considered as the best estimate of the phylogeny of subgenus *Avaritia* based on our dataset. Figure 3 shows the BI tree resulting from the phylogenetic analysis of the combined dataset according to the strategy P2, i.e. one partition for the three mitochondrial genes and one partition for the two nuclear markers.

With the exception of the position of *C.* *dewulfi*, *C.* *jacobsoni*, *C.* *brevipalpis* and *C.* *kibatiensis*, the major part of MP, BI and ML topologies were congruent. In the resulting topology (Fig. 3), most nodes were supported by cpp-values above 91%. The Obsoletus, Imicola and Grahamii groups were all supported by cpp-values of 99–100%. Furthermore, the BI analysis provided support for the existence of the Boophagus group by a cpp-value of 91%. The monophyletic status of the Obsoletus and Imicola groups was strongly supported with cpp-values of 100% and 99%, respectively. Within the Imicola group, the recently described species *C.* *asiana* Bellis [30] was highly supported (cpp of 100%) as very close to the Australian species *C.* *brevitarsis*. The clade containing *C.* *orientalis* Macfie, *C.* *fulvus* Sen & Das Gupta*, C.* *tainanus* Kieffer and *C.* *obscurus* Tokunaga & Murachi was not strongly supported (cpp of 65%). The placement of *C.* *gulbenkiani* with either *C.* *tororoensis* or *C.* *brevipalpis* was not supported in this study.

## Discussion

To our knowledge, this is the first systematic study of *Culicoides* (*Avaritia*) based on a five-marker phylogenetic approach and including species from all biogeographical regions. Among the six genetic markers studied, the mitochondrial *cox*2 and *cytb* markers both showed strong phylogenetic signal to separate closely related species but were less informative for basal topology. The combined non-coding region ITS1+ITS2 showed to be useful to describe basal tree topologies but *5.8S* was entirely uninformative. The *cox*1 region supported both basal topology and the discrimination of closely related species. *cox*2, newly tested for intra-subgeneric systematic research, provided informative knowledge of in-group relationships for closely related clades.

### Phylogenetic relationships within subgenus *Avaritia*

Combined phylogenetic information from several distinct genes has provided some insight on the relationships within the subgenus and between species groups.

With the exception of *C.* *kanagai*, the species studied which have the combination of cell r_2_ dark to apex and hairy eyes, including *C.* *actoni*, *C.* *grahamii* and *C.* *pusillus*, were found to be phylogenetically clustered in the Grahamii group. Despite its closer morphological similarity to species of the Grahamii group, our molecular analysis placed *C.* *kanagai* closer to *C.* *tororoensis* and *C.* *gulbenkiani* than to the Grahamii group clade. *Culicoides tororoensis* and *C.* *gulbenkiani*, however, differ morphologically from *C.* *kanagai* as they are large species, lack interfacetal hairs and have three pale spots in the third radial cell. In the Oriental region, *C.* *hui* has this latter feature but differs as cell r_2_ is pale apically. Both Wirth & Hubert [9] and Meiswinkel [11] placed *C.* *hui* but not *C.* *tororoensis* or *C.* *gulbenkiani*, into the Orientalis group but none of these species were placed close to *C.* *orientalis* in any of our phylogenetic reconstructions. Further investigations to unravel the relationships within the Orientalis group would be necessary including species such as *C.* *hui*, *C.* *flavipunctatus* Kitaoka, *C.* *dumdumi* Sen & Das Gupta*, C.* *brosseti* Vattier & Adam, *C.* *dubitatus* Kremer, Rebholtz-Hirtzel & Delécolle and *C.* *trifasciellus*. Bakhoum et al. [43] in their comprehensive phylogenetic study of the Afrotropical species, included *C.* *trifasciellus* and the putative new species *Culicoides* sp. #20 into the Orientalis group. The phylogenetic data of species morphologically affiliated to the Orientalis group *sensu* Meiswinkel [11] are currently sparse and a comprehensive integrative taxonomic study of this group is required.

Morphological feature variations are often observed in the genus *Culicoides* especially for species with large spatial distribution. Similarly, genetic variations were observed in our dataset for species present across the Oriental and Australasian regions (*C.* *actoni*, *C.* *fulvus*, *C.* *brevipalpis* and *C.* *jacobsoni*) and could be indicative of cryptic diversity or on-going speciation. Gopurenko et al. [42] reported similar cryptic diversity for *C.* *actoni*, *C.* *brevipalpis* and *C.* *jacobsoni* while *C.* *fulvus* appeared to be genetically uniform across Australasia and East Asia. Similarly, our study showed genetic diversity between *C.* *bolitinos* specimens from South Africa and La Reunion/Madagascar which is consistent with observations of recent studies [43, 59]. These *C.* *bolitinos* populations showed genetic distances similar to that between *C.* *brevitarsis* and *C.* *asiana* supporting the existence of a cryptic species of *C.* *bolitinos.* Further studies incorporating specimens from populations across the full distribution of this species are required to clarify the status of this species.

Species of Andicola group are endemic to high altitudes from 1600 to 3200 m in the central and western ranges of the Andes [27, 28]. Due to their rarity and difficulty to access, we were unable to secure any specimens belonging to Andicola group for inclusion in this study. Consequently, the monophyly and placement of this group within the subgenus remains untested.

### Systematic revision

The status of the Obsoletus group is supported by molecular analysis to include the following 8 species: *C.* *abchazicus* Dzhafarov, *C.* *alachua* Jamnback & Wirth*, C.* *chiopterus* (Meigen), *C.* *montanus* Shakirzjanova, *C.* *obsoletus*, *C.* *sanguisuga* (Coquillett), *C.* *scoticus* Downes & Kettle and *C.* *sinanoensis. Culicoides dewulfi* has a wing pattern consistent with members of the Obsoletus group but has male genitalia more similar to those in species of the Imicola group which has led to conflicting opinions about its placement within this group [10, 34]. In this study, *C.* *dewulfi* was clearly phylogenetically separate from any group, including the Obsoletus and Imicola groups, which agrees with the conclusions of other studies [15, 60]. Evidence of cryptic species diversity within *C.* *obsoletus* has been reported previously from Sweden [61], Switzerland [62] and the UK [63] but was not supported in our analysis of specimens from France, Norway, Spain and the USA. *Culicoides gornostaevae* Mirzaeva, a species recorded from Russia to Japan, was recently recorded in Norway, Poland and Sweden [64] but the latter study did not investigate the *cox*1 marker and whether these specimens morphologically identified as *C.* *gornostaevae* fit with one of the genetic forms of *C.* *obsoletus* remain to be investigated.

The monophyly of the Imicola group within subgenus *Avaritia* was well supported in the present analyses, in agreement with previous reports [22, 35]. The placement of all species into the Imicola group by Meiswinkel [11] and Bellis et al. [30], i.e. *C.* *asiana*, *C.* *bolitinos*, *C.* *brevitarsis*, *C.* *imicola*, *C.* *kwagga*, *C.* *loxodontis* Meiswinkel, *C.* *miombo* Meiswinkel, *C.* *nudipalpis* Delfinado, *C.* *pseudopallidipennis* Clastrier and *C.* *tuttifrutti* Meiswinkel, Cornet & Dyce, were supported in the present study. Based on the geographical origin of the specimens studied, we therefore report here *C.* *asiana* as a new record for Vietnam and *C.* *imicola* as a new record for Taiwan.

In their comprehensive study of the *Culicoides* from Southeast Asia, Wirth & Hubert [17] mention the morphological similarity between the Actoni group, comprising the Oriental and Australian species *C.* *actoni* and *C.* *minimus*, and the Pusillus group of Wirth et al. [29]. Their assessment combined with our phylogenetic results suggest merging the Actoni and Pusillus groups into the Grahamii group, this name having precedence as *C.* *grahamii* is the oldest described member of the group. The placement of *C.* *minimus*, *C.* *pusilloides* and *C.* *impusilloides* remains untested as these were not included in this study but their previous placement into the Actoni and Pusillus groups based on morphological evidence suggests they are related and so we place them tentatively into the Grahamii group.

Despite somewhat relatively weak support, the Boophagus group of Dyce et al. [17] (= Suzukii group of Meiswinkel et al. [16]) including *C.* *boophagus* Macfie, *C.* *suzukii* Kitaoka *sensu* Lien et al. [46] and *C.* *wadai* appears to be a valid group. Morphologically, we define this group as having a quadrate dark area at the tip of vein M_2_ surrounded by a pale area. However, the illustrations of *C.* *suzukii* provided by Kitaoka show the dark area covering the vein M2 being continuous to the dark quadrate area on tip of the vein M2 [45, 65, 66] which differs to the specimen illustrated by Lien et al. [46]. The specimen from Taiwan examined by us is morphologically consistent with *C.* *suzukii sensu* Lien et al. [46], and until the discrepancies between these interpretations of this species are clarified, the position of “true” *C.* *suzukii sensu* Kitaoka [45] within the Boophagus group remains unknown.

First mentioned by Dyce et al. [19] and Dyce [31], the Jacobsoni group was composed of two species, one of which is undescribed, until an integrative taxonomic study incorporating *cox*1 and *CAD* analysis by Gopurenko et al. [42] revealed the existence of a further four undescribed species making a total of six species. These authors also provided evidence for the monophyly of the Jacobsoni group. Even though our study has included only two of the cryptic species reported by Gopurenko et al. [42], our results would provide some support to the monophyly of the Jacobsoni group.

## Conclusions

The combined use of five distinct molecular markers belonging to nuclear (ITS1 and ITS2) and mitochondrial DNA (*cox*1, *cox*2 and *cytb*) provided meaningful insights in relation to taxonomic studies and provided direction for future studies. Newly studied for *Culicoides* phylogeny, the *cox*2 gene appeared to be useful to investigate closely related species. On the opposite, the *5.8S* marker was highly conserved and uninformative. The groups mentioned in this study represented monophyletic clades and the taxonomic rearrangement proposed was based on phylogenetic evidence. Within subgenus *Avaritia*, the Obsoletus group was sister to all other groups included in this study. The Imicola group was phylogenetically robust, morphologically well defined, and a cryptic diversity within *C.* *bolitinos* remains to be investigated. Similarly, cryptic diversity was identified in this study within *C.* *actoni*, *C.* *brevipalpis*, *C.* *fulvus* and *C.* *jacobsoni*, which opens an entire field of investigation. The Actoni, Pusillus and Grahamii groups have been merged and some support was provided for the Boophagus and Jacobsoni groups. The monophyly and composition of the Orientalis group remains unclear and further investigations would be necessary to improve its definition. This study at a global scale, has allowed a better definition of the subgenus *Culicoides* (*Avaritia*) and as such it constitutes a step forward towards a global reassessment of the systematic and phylogenetics of the genus *Culicoides*.

## Supplementary information

**Additional file 1: Table S1.** List of specimens included in this study, along with their identification numbers (voucher code), countries and GenBank accession numbers for each DNA region.

## Data Availability

The datasets used during the current study are available from the corresponding author upon reasonable request.
