# Supplementary material for: A phylogenetic analysis of the biting midges belonging to Culicoides Latreille (Diptera: Ceratopogonidae) subgenus Avaritia using molecular data
Source: Parasit Vectors. 2020 May 12;13:243. doi: 10.1186/s13071-020-04111-4 (PMC7216621; doi:10.1186/s13071-020-04111-4)
Supplement: Supplementary file 1 — Additional file 1: Table S1. List of specimens included in this study, along with their identification numbers (voucher code), countries and GenBank accession numbers for each DNA region. [file 13071_2020_4111_MOESM1_ESM.pdf]

Additional file 1: Table S1.

List of specimens included in this study, along with their identification numbers (voucher code), countries and Genbank accession numbers for each DNA region. *rDNA* referred to sequences available for the ITS1-5.8S-ITS2 region.

Species	Country	Voucher	COI	COII	Cytb	rDNA	Collector	Determinator	Reference
C. (A.) abchazicus	France	FRXY01	MK760081	MK860204	MK860305	MK892988	C. Ninio	J.C. Delécolle	This study
C. (A.) abchazicus	France	FRXY02	MK760082	MK860205	-	MK892989	C. Ninio	J.C. Delécolle	This study
C. (A.) actoni	Australia	AUBR04	MK760083	-	MK860306	MK892990	N. Hunt	G. Bellis	This study
C. (A.) actoni	Australia	AUBR05	MK760084	-	MK860307	MK892991	N. Hunt	G. Bellis	This study
C. (A.) actoni	Taïwan	TWSN01	MK760085	-	-	MK892992	M.H. Weng	J.C. Lien	This study
C. (A.) actoni	Taïwan	TWSN02	MK760086	-	-	-	M.H. Weng	J.C. Lien	This study
C. (A.) actoni	Taïwan	TWSN03	MK760087	-	MK860308	-	M.H. Weng	J.C. Lien	This study
C. (A.) alachua	United States (East)	USLN01	MK760088	-	MK860309	MK892993	D.A. Swanson	D.A. Swanson	This study
C. (A.) asiana	Vietnam	VNMC23	MK760107	-	MK860323	-	Le Duc Do	B. Mathieu	This study
C. (A.) bolitinos	Madagascar	MGBN04	MK760089	-	MK860310	-	V. Robert	B. Mathieu	This study
C. (A.) bolitinos	Reunion island	REBG01	MK760090	-	MK860311	-	A. Desvars	J.C. Delécolle	This study
C. (A.) bolitinos	Reunion island	REBG09	MK760091	-	MK860312	-	A. Desvars	J.C. Delécolle	This study
C. (A.) bolitinos	Reunion island	REBG10	MK760092	MK860206	MK860313	-	A. Desvars	J.C. Delécolle	This study
C. (A.) bolitinos	Reunion island	REBG11	MK760093	-	MK860314	-	A. Desvars	J.C. Delécolle	This study
C. (A.) bolitinos	South Africa	ZAKW01	MK760094	MK860207	MK860315	-	-	K. Labuschagne	This study
C. (A.) bolitinos	South Africa	ZAKW02	MK760095	MK860208	MK860316	-	-	K. Labuschagne	This study
C. (A.) bolitinos	South Africa	ZAKW05	MK760096	MK860209	MK860317	-	-	K. Labuschagne	This study
C. (A.) boophagus	Papua New Guinea	PGKR01	MK760097	MK860210	MK860318	MK892994	P. Boland	G. Bellis	This study
C. (A.) brevipalpis	Australia	AUKL01	MK760098	MK860211	-	MK892995	M. Keane	G. Bellis	This study
C. (A.) brevipalpis	Australia	AUKL02	MK760099	MK860212	-	-	M. Keane	G. Bellis	This study

C. (A.) brevipalpis	Australia	AUKL04	MK760100	MK860213	-	MK892996	M. Keane	G. Bellis	This study
C. (A.) brevipalpis	Australia	AUKL05	MK760101	MK860214	MK860319	MK892997	M. Keane	G. Bellis	This study
C. (A.) brevitarsis	Australia	AUBR01	MK760102	MK860215	-	MK892998	N. Hunt	G. Bellis	This study
C. (A.) brevitarsis	Australia	AUBR02	MK760103	MK860216	MK860320	MK892999	N. Hunt	G. Bellis	This study
C. (A.) brevitarsis	Australia	AUBR03	MK760104	MK860217	-	-	N. Hunt	G. Bellis	This study
C. (A.) brevitarsis	Australia	AUBR08	MK760105	MK860218	MK860321	MK893000	N. Hunt	G. Bellis	This study
C. (A.) brevitarsis	Australia	AUBR09	MK760106	MK860219	MK860322	-	N. Hunt	G. Bellis	This study
C. (A.) chiopterus	France	FRPS28	MK760108	MK860220	-	MK893001	S. Mutel	B. Mathieu	This study
C. (A.) chiopterus	France	FRPS81	MK760109	MK860221	-	MK893002	E. Bois-Kuentz	B. Mathieu	This study
C. (A.) chiopterus	France	FRVN01	MK760110	MK860222	-	-	Costet-Cherifi	B. Mathieu	This study
C. (A.) chiopterus	Russia	RUKR02	MK760111	-	-	-	N. Brodskaia	N. Brodskaia	This study
C. (A.) dewulfi	Spain	ESBR01	MK760112	MK860223	MK860324	MK893003	N. Pagès	N. Pagès	This study
C. (A.) dewulfi	Spain	ESBR02	MK760113	MK860224	MK860325	-	N. Pagès	N. Pagès	This study
C. (A.) dewulfi	Spain	ESBR03	MK760114	MK860225	MK860326	-	N. Pagès	N. Pagès	This study
C. (A.) dewulfi	France	FRFN01	MK760115	MK860226	MK860327	MK893004	J. Gosset	B. Mathieu	This study
C. (A.) dewulfi	France	FRMN01	MK760116	MK860227	MK860328	MK893005	S. Rondeau	B. Mathieu	This study
C. (A.) dewulfi	France	FRPS68	-	MK860228	MK860329	-	M. Pizard	B. Mathieu	This study
C. (A.) fulvus	Australia	AUBR06	MK760117	-	MK860330	MK893006	N. Hunt	G. Bellis	This study
C. (A.) fulvus	Australia	AUBR07	MK760118	-	MK860331	MK893007	N. Hunt	G. Bellis	This study
C. (A.) fulvus	Vietnam	VNMC09	MK760119	-	MK860332	-	Le Duc Do	B. Mathieu	This study
C. (A.) fulvus	Vietnam	VNMC10	MK760120	-	MK860333	-	Le Duc Do	B. Mathieu	This study
C. (A.) fulvus	Vietnam	VNMC11	MK760121	-	MK860334	-	Le Duc Do	B. Mathieu	This study
C. (A.) fulvus	Vietnam	VNMC17	MK760122	-	MK860335	-	Le Duc Do	B. Mathieu	This study
C. (A.) fulvus	Vietnam	VNMC24	MK760123	-	MK860336	-	Le Duc Do	B. Mathieu	This study
C. (A.) grahamii	Reunion island	REBG04	MK760124	-	-	-	A. Desvars	J.C. Delécolle	This study
C. (A.) grahamii	Reunion island	REBG05	MK760125	-	-	MK893008	A. Desvars	J.C. Delécolle	This study

C. (A.) grahamii	Reunion island	RECL01	MK760126	-	MK860337	-	A. Desvars	J.C. Delécolle	This study
C. (A.) grahamii	Reunion island	RESR03	MK760127	-	-	MK893009	A. Desvars	J.C. Delécolle	This study
C. (A.) gulbenkiani	South Africa	ZAGR01	MK760128	MK860229	MK860338	MK893010	-	K. Labuschagne	This study
C. (A.) gulbenkiani	South Africa	ZAGR02	MK760129	MK860230	MK860339	MK893011	-	K. Labuschagne	This study
C. (A.) gulbenkiani	South Africa	ZAGR03	MK760130	-	MK860340	MK893012	-	K. Labuschagne	This study
C. (A.) gulbenkiani	South Africa	ZAGR04	MK760131	-	MK860341	MK893013	-	K. Labuschagne	This study
C. (A.) gulbenkiani	South Africa	ZAGR05	MK760132	-	MK860342	-	-	K. Labuschagne	This study
C. (A.) imicola	Burkina faso	BFBB03	MK760133	MK860231	MK860343	-	F. Simard	B. Mathieu	This study
C. (A.) imicola	Benin	BJTR04	MK760134	MK860232	MK860344	-	T. Baldet	B. Mathieu	This study
C. (A.) imicola	Benin	BJTR05	MK760135	MK860233	MK860345	-	T. Baldet	B. Mathieu	This study
C. (A.) imicola	Benin	BJTR06	MK760136	MK860234	MK860346	-	T. Baldet	B. Mathieu	This study
C. (A.) imicola	Benin	BJTR12	MK760137	MK860235	MK860347	-	T. Baldet	B. Mathieu	This study
C. (A.) imicola	Cameroon	CMGB01	MK760138	MK860236	MK860348	-	C. Cêtre-Sossah	B. Mathieu	This study
C. (A.) imicola	Cameroon	CMGB02	MK760139	MK860237	MK860349	-	C. Cêtre-Sossah	B. Mathieu	This study
C. (A.) imicola	Cameroon	CMGB03	MK760140	MK860238	MK860350	-	C. Cêtre-Sossah	B. Mathieu	This study
C. (A.) imicola	Cameroon	CMGB04	MK760141	MK860239	MK860351	-	C. Cêtre-Sossah	B. Mathieu	This study
C. (A.) imicola	Cameroon	CMGB06	MK760142	MK860240	MK860352	-	C. Cêtre-Sossah	B. Mathieu	This study
C. (A.) imicola	Algeria	DZBZ03	MK760143	MK860241	MK860353	-	M. Djerbal	J.C. Delécolle	This study
C. (A.) imicola	Algeria	DZBZ04	MK760144	MK860242	MK860354	-	M. Djerbal	J.C. Delécolle	This study
C. (A.) imicola	Ethiopia	ETFR01	MK760145	MK860243	-	MK893014	T. Baldet	B. Mathieu	This study
C. (A.) imicola	Ethiopia	ETFR02	-	MK860244	MK860355	-	T. Baldet	B. Mathieu	This study
C. (A.) imicola	Ethiopia	ETFR03	MK760146	MK860245	MK860356	-	T. Baldet	B. Mathieu	This study
C. (A.) imicola	Ethiopia	ETFR04	MK760147	MK860246	MK860357	-	T. Baldet	B. Mathieu	This study
C. (A.) imicola	Ethiopia	ETFR05	MK760148	MK860247	-	-	T. Baldet	B. Mathieu	This study
C. (A.) imicola	Ethiopia	ETRM01	MK760149	MK860248	MK860358	MK893015	T. Baldet	B. Mathieu	This study
C. (A.) imicola	Ethiopia	ETRM02	MK760150	-	MK860359	-	T. Baldet	B. Mathieu	This study

C. (A.) imicola	Ethiopia	ETRM03	MK760151	MK860249	MK860360	-	T. Baldet	B. Mathieu	This study
C. (A.) imicola	Ethiopia	ETRM07	MK760152	MK860250	-	-	T. Baldet	B. Mathieu	This study
C. (A.) imicola	Ethiopia	ETRM08	MK760153	MK860251	-	-	T. Baldet	B. Mathieu	This study
C. (A.) imicola	France	FRPS17	-	-	MK860361	-	G. Bousquet	B. Mathieu	This study
C. (A.) imicola	France	FRPS18	MK760154	-	MK860362	MK893016	G. Bousquet	B. Mathieu	This study
C. (A.) imicola	France	FRPS20	MK760155	MK860252	MK860363	-	G. Bousquet	B. Mathieu	This study
C. (A.) imicola	France	FRPS21	-	MK860253	MK860364	-	B. Mathieu	B. Mathieu	This study
C. (A.) imicola	France	FRRQ01	MK760156	MK860254	-	-	B. Mathieu	B. Mathieu	This study
C. (A.) imicola	Madagascar	MGBN01	MK760157	MK860255	MK860365	-	V. Robert	B. Mathieu	This study
C. (A.) imicola	Madagascar	MGBN02	MK760158	MK860256	MK860366	-	V. Robert	B. Mathieu	This study
C. (A.) imicola	Madagascar	MGBN08	MK760159	MK860257	MK860367	-	V. Robert	B. Mathieu	This study
C. (A.) imicola	Mauritius	MUMD01	MK760160	MK860258	-	-	F. Biteau-Coroller	J.C. Delécolle	This study
C. (A.) imicola	Mauritius	MUMD02	MK760161	MK860259	MK860368	-	F. Biteau-Coroller	J.C. Delécolle	This study
C. (A.) imicola	Mauritius	MUMD04	MK760162	MK860260	MK860369	-	F. Biteau-Coroller	J.C. Delécolle	This study
C. (A.) imicola	Mauritius	MUPS23	-	MK860261	MK860370	-	F. Biteau-Coroller	J.C. Delécolle	This study
C. (A.) imicola	Reunion island	RESP01	MK760163	MK860262	-	-	A. Desvars	J.C. Delécolle	This study
C. (A.) imicola	Reunion island	RESP02	MK760164	MK860263	MK860371	-	A. Desvars	J.C. Delécolle	This study
C. (A.) imicola	Reunion island	RESP04	MK760165	MK860264	MK860372	-	A. Desvars	J.C. Delécolle	This study
C. (A.) imicola	Reunion island	RESP05	MK760166	MK860265	MK860373	-	A. Desvars	J.C. Delécolle	This study
C. (A.) imicola	South Africa	ZAPR01	MK760167	MK860266	MK860374	-	-	K. Labuschagne	This study
C. (A.) imicola	South Africa	ZAPR03	MK760168	MK860267	MK860375	-	-	K. Labuschagne	This study
C. (A.) imicola	South Africa	ZAPR04	MK760169	MK860268	-	-	-	K. Labuschagne	This study
C. (A.) imicola	South Africa	ZAPR05	MK760170	MK860269	MK860376	-	-	K. Labuschagne	This study
C. (A.) jacobsoni	Taïwan	TWSN05	MK760171	-	MK860377	-	M.H. Weng	J.C. Lien	This study
C. (A.) jacobsoni	Taïwan	TWSN06	MK760172	-	MK860378	-	M.H. Weng	J.C. Lien	This study
C. (A.) jacobsoni	Vietnam	VNMC01	MK760173	-	MK860379	MK893017	Le Duc Do	B. Mathieu	This study

C. (A.) jacobsoni	Vietnam	VNMC02	MK760174	-	MK860380	-	Le Duc Do	B. Mathieu	This study
C. (A.) jacobsoni	Vietnam	VNMC03	MK760175	MK860270	MK860381	-	Le Duc Do	B. Mathieu	This study
C. (A.) jacobsoni	Vietnam	VNMC19	MK760176	-	MK860382	-	Le Duc Do	B. Mathieu	This study
C. (A.) jacobsoni	Vietnam	VNMC20	MK760177	-	MK860383	-	Le Duc Do	B. Mathieu	This study
C. (A.) jacobsoni	Vietnam	VNMC21	MK760178	-	MK860384	-	Le Duc Do	B. Mathieu	This study
C. (A.) jacobsoni	Vietnam	VNMC22	MK760179	-	MK860385	-	Le Duc Do	B. Mathieu	This study
C. (A.) kanagai	South Africa	ZAKR07	MK760180	-	MK860386	MK893018	-	K. Labuschagne	This study
C. (A.) kibatiensis	Reunion island	REBG06	MK760181	-	MK860387	-	A. Desvars	J.C. Delécolle	This study
C. (A.) kibatiensis	Reunion island	REBG07	MK760182	-	-	MK893019	A. Desvars	J.C. Delécolle	This study
C. (A.) kibatiensis	Reunion island	REBG08	MK760183	-	MK860388	-	A. Desvars	J.C. Delécolle	This study
C. (A.) kibatiensis	Reunion island	RESL01	MK760184	-	MK860389	MK893020	A. Desvars	J.C. Delécolle	This study
C. (A.) kibatiensis	Reunion island	RESL02	MK760185	-	MK860390	MK893021	A. Desvars	J.C. Delécolle	This study
C. (A.) kibatiensis	Reunion island	RESL03	MK760186	-	MK860391	-	A. Desvars	J.C. Delécolle	This study
C. (A.) kibatiensis	Reunion island	RESL04	MK760187	-	MK860392	-	A. Desvars	J.C. Delécolle	This study
C. (A.) loxodontis	South Africa	ZAKR01	MK760188	MK860271	-	-	-	K. Labuschagne	This study
C. (A.) loxodontis	South Africa	ZAKR02	MK760189	MK860272	-	-	-	K. Labuschagne	This study
C. (A.) loxodontis	South Africa	ZAKR03	MK760190	MK860273	-	-	-	K. Labuschagne	This study
C. (A.) loxodontis	South Africa	ZAKR11	MK760191	MK860274	-	MK893022	-	K. Labuschagne	This study
C. (A.) miombo	Benin	BJTR02	MK760192	MK860275	MK860393	MK893023	T. Baldet	B. Mathieu	This study
C. (A.) miombo	Benin	BJTR08	MK760193	MK860276	MK860394	-	T. Baldet	B. Mathieu	This study
C. (A.) miombo	Benin	BJTR09	MK760194	MK860277	MK860395	-	T. Baldet	B. Mathieu	This study
C. (A.) miombo	Benin	BJTR11	MK760195	-	MK860396	-	T. Baldet	B. Mathieu	This study
C. (A.) miombo	Cameroon	CMGB07	MK760196	MK860278	MK860397	-	C. Cêtre-Sossah	B. Mathieu	This study
C. (A.) miombo	Cameroon	CMGB09	MK760197	MK860279	MK860398	-	C. Cêtre-Sossah	B. Mathieu	This study
C. (A.) miombo	Cameroon	CMGB10	MK760198	MK860280	MK860399	-	C. Cêtre-Sossah	B. Mathieu	This study
C. (A.) miombo	Cameroon	CMGB11	MK760199	MK860281	MK860400	-	C. Cêtre-Sossah	B. Mathieu	This study

C. (A.) miombo	Madagascar	MGNR03	MK760200	-	-	-	L. Tantely	B. Mathieu	This study
C. (A.) miombo	Madagascar	MGNR04	-	-	MK860401	-	L. Tantely	B. Mathieu	This study
C. (A.) miombo	Madagascar	MGXX01	-	-	MK860402	MK893024	L. Tantely	B. Mathieu	This study
C. (A.) montanus	Algeria	DZTZ01	MK760201	-	MK860403	-	M. Djerbal	J.C. Delécolle	This study
C. (A.) montanus	France	FRPS29	-	-	MK860404	MK893025	G. Bousquet	J.C. Delécolle	This study
C. (A.) montanus	France	FRXX01	MK760202	-	MK860405	-	G. Bousquet	J.C. Delécolle	This study
C. (A.) montanus	France	FRXX02	MK760203	-	MK860406	MK893026	H. Guis	J.C. Delécolle	This study
C. (A.) montanus	France	FRXX03	MK760204	-	MK860407	-	H. Guis	J.C. Delécolle	This study
C. (A.) montanus	Italy	ITLG01	MK760205	MK860282	MK860408	MK893027	M. Goffredo	M. Goffredo	This study
C. (A.) montanus	Italy	ITLG02	MK760206	MK860283	MK860409	MK893028	M. Goffredo	M. Goffredo	This study
C. (A.) montanus	Italy	ITLG03	MK760207	MK860284	MK860410	-	M. Goffredo	M. Goffredo	This study
C. (A.) nudipalpis	Taïwan	TWKN01	MK760208	-	MK860411	-	J.C. Lien	J.C. Lien	This study
C. (A.) nudipalpis	Taïwan	TWKN02	MK760209	-	-	-	J.C. Lien	J.C. Lien	This study
C. (A.) obscurus	Australia	AUMN01	MK760210	MK860285	-	MK893029	J. Anderson	G. Bellis	This study
C. (A.) obscurus	Australia	AUMN02	MK760211	MK860286	-	MK893030	J. Anderson	G. Bellis	This study
C. (A.) obscurus	Australia	AUMN03	MK760212	MK860287	-	MK893031	J. Anderson	G. Bellis	This study
C. (A.) obsoletus	Spain	ESBR04	MK760213	-	MK860412	-	N. Pagès	N. Pagès	This study
C. (A.) obsoletus	Spain	ESBR05	MK760214	-	MK860413	-	N. Pagès	N. Pagès	This study
C. (A.) obsoletus	France	FRPS08	MK760215	-	-	MK893032	M. Pizard	B. Mathieu	This study
C. (A.) obsoletus	Algeria	FRPS25	-	-	MK860414	-	M. Djerbal	J.C. Delécolle	This study
C. (A.) obsoletus	France	FRPS69	MK760216	-	MK860415	MK893033	M. Demol	B. Mathieu	This study
C. (A.) obsoletus	France	FRPS71	MK760217	-	MK860416	MK893034	P. Catez	B. Mathieu	This study
C. (A.) obsoletus	France	FRPS73	MK760218	-	MK860417	MK893035	C. Herisson	B. Mathieu	This study
C. (A.) obsoletus	France	FRPS75	MK760219	-	MK860418	-	P. Catez	B. Mathieu	This study
C. (A.) obsoletus	Norway	NOXX01	MK760220	MK860288	MK860419	MK893036	F. Schaffner	B. Mathieu	This study
C. (A.) obsoletus	Norway	NOXX02	MK760221	-	MK860420	-	F. Schaffner	B. Mathieu	This study

C. (A.) obsoletus	Norway	NOXX03	-	-	MK860421	-	F. Schaffner	B. Mathieu	This study
C. (A.) obsoletus	United States (East)	USPC01	MK760222	MK860289	MK860422	MK893037	D. Swanson	D. Swanson	This study
C. (A.) orientalis	Vietnam	VNMC12	MK760223	MK860290	-	-	Le Duc Do	B. Mathieu	This study
C. (A.) orientalis	Vietnam	VNMC14	MK760224	-	-	-	Le Duc Do	B. Mathieu	This study
C. (A.) orientalis	Vietnam	VNMC16	MK760225	-	-	-	Le Duc Do	B. Mathieu	This study
C. (A.) orientalis	Vietnam	VNMC25	MK760226	-	-	-	Le Duc Do	B. Mathieu	This study
C. (A.) orientalis	Vietnam	VNMC29	MK760227	-	-	-	Le Duc Do	B. Mathieu	This study
C. (A.) orientalis	Vietnam	VNMC30	MK760228	MK860291	MK860423	-	Le Duc Do	B. Mathieu	This study
C. (A.) pseudopallidipennis	Benin	BJTR13	MK760229	MK860292	-	MK893038	T. Baldet	B. Mathieu	This study
C. (A.) pseudopallidipennis	Benin	BJTR14	MK760230	MK860293	MK860424	-	T. Baldet	B. Mathieu	This study
C. (A.) pseudopallidipennis	Benin	BJTR17	MK760231	-	MK860425	-	T. Baldet	B. Mathieu	This study
C. (A.) pusillus	French Guyana	GFMC01	MK760232	-	MK860426	MK893039	I. Dusfour	B. Mathieu	This study
C. (A.) pusillus	French Guyana	GFMC02	MK760233	MK860294	MK860427	MK893040	I. Dusfour	B. Mathieu	This study
C. (A.) pusillus	Guadeloupe	GPSL01	MK760234	-	-	MK893041	J.C. Delécolle	J.C. Delécolle	This study
C. (A.) pusillus	Guadeloupe	GPSL02	MK760235	-	-	-	J.C. Delécolle	J.C. Delécolle	This study
C. (A.) pusillus	Guadeloupe	GPSL03	MK760236	-	-	-	J.C. Delécolle	J.C. Delécolle	This study
C. (A.) sanguisuga	United States (East)	USRC01	MK760237	-	MK860428	MK893042	D. Swanson	D. Swanson	This study
C. (A.) sanguisuga	United States (East)	USRC02	MK760238	MK860295	MK860429	MK893043	D. Swanson	D. Swanson	This study
C. (A.) scoticus	Spain	ESBR06	-	MK860296	-	MK893044	N. Pagès	N. Pagès	This study
C. (A.) scoticus	Spain	ESBR07	-	MK860297	-	-	N. Pagès	N. Pagès	This study
C. (A.) scoticus	France	FRPS70	-	MK860298	-	-	P. Decroocq	B. Mathieu	This study
C. (A.) scoticus	France	FRPS72	MK760239	MK860299	MK860430	MK893045	P. Catez	B. Mathieu	This study
C. (A.) scoticus	France	FRPS74	MK760240	MK860300	-	-	C. Herisson	B. Mathieu	This study
C. (A.) sinanoensis	Russia	RUXX01	MK760241	-	MK860431	MK893046	V. Glukhova	V. Glukhova	This study
C. (A.) sinanoensis	Russia	RUXX02	MK760242	-	MK860432	MK893047	V. Glukhova	V. Glukhova	This study
C. (A.) sinanoensis	Russia	RUXX03	MK760243	-	MK860433	-	V. Glukhova	V. Glukhova	This study

C. (A.) sinanoensis	Russia	RUXX04	MK760244	-	-	-	V. Glukhova	V. Glukhova	This study
C. (A.) suzukii	Vietnam	VNMC07	MK760245	-	-	-	Le Duc Do	B. Mathieu	This study
C. (A.) tainanus	Taïwan	TWSN07	-	-	MK860434	-	M.H. Weng	J.C. Lien	This study
C. (A.) tainanus	Taïwan	TWSN08	MK760246	-	MK860435	-	M.H. Weng	J.C. Lien	This study
C. (A.) tainanus	Taïwan	TWSN09	MK760247	-	MK860436	-	M.H. Weng	J.C. Lien	This study
C. (A.) tororoensis	South Africa	ZAKR04	MK760248	-	-	MK893048	-	K. Labuschagne	This study
C. (A.) tororoensis	South Africa	ZAKR09	MK760249	-	-	MK893049	-	K. Labuschagne	This study
C. (A.) tororoensis	South Africa	ZAKR10	MK760250	-	-	-	-	K. Labuschagne	This study
C. (A.) tuttifrutti	South Africa	ZAPR06	MK760251	MK860301	MK860437	MK893050	-	K. Labuschagne	This study
C. (A.) wadai	Australia	AUDG01	MK760252	MK860302	MK860438	MK893051	N. Hunt	G. Bellis	This study
C. (A.) wadai	Australia	AUDG02	MK760253	MK860303	MK860439	MK893052	N. Hunt	G. Bellis	This study
C. (A.) wadai	Australia	AUDG03	MK760254	MK860304	MK860440	MK893053	N. Hunt	G. Bellis	This study
C. (A.) actoni	Japan	-	-	-	-	AB462259	-	-	Matsumoto et al. [41]
C. (A.) actoni	Australia	ww08186	KJ162953	-	-	-	-	-	Bellis et al. [30]
C. (M.) arakawae	-	-	NC009809	NC009809	NC009809	AJ489503	-	-	Matsumoto et al. [37], Li et al. [68]
C. (A.) asiana	Laos	ww06258	KJ162954	-	-	-	-	-	Bellis et al. [30]
C. (A.) asiana	Japan	ww08443	KJ162955	-	-	-	-	-	Bellis et al. [30]
C. (A.) asiana	Japan	ww08444	KJ162956	-	-	-	-	-	Bellis et al. [30]
C. (A.) asiana	Japan	ww08438	KJ162957	-	-	-	-	-	Bellis et al. [30]
C. (A.) asiana	Japan	ww08437	KJ162958	-	-	-	-	-	Bellis et al. [30]
C. (A.) asiana	East Timor	ww06007	KJ162959	-	-	-	-	-	Bellis et al. [30]
C. (A.) asiana	Japan	ww08439	KJ162960	-	-	-	-	-	Bellis et al. [30]
C. (A.) bolitinos	South Africa	-	AF071929	-	-	-	-	-	Linton et al. [13]
C. (A.) bolitinos	South Africa	ww14302	KJ162960	-	-	-	-	-	Bellis et al. [30]
C. (A.) bolitinos	South Africa	ww14302	KJ162962	-	-	-	-	-	Bellis et al. [30]

C. (A.) brevipalpis	Japan	-	AB360998	AB360998	-	-	-	-	Matsumoto et al. [37]
C. (A.) brevipalpis	Japan	ww09012	KJ162963	-	-	-	-	-	Bellis et al. [30]
C. (A.) brevipalpis	Japan	ww09013	KJ162964	-	-	-	-	-	Bellis et al. [30]
C. (A.) brevipalpis	Japan	ww09014	KJ162965	-	-	-	-	-	Bellis et al. [30]
C. (A.) brevitarsis	Japan	-	AB360995	AB360995	-	-	-	-	Matsumoto et al. [37]
C. (A.) brevitarsis	China	ww06255	KJ162966	-	-	-	-	-	Bellis et al. [30]
C. (A.) brevitarsis	East Timor	ww05961	KJ162968	-	-	-	-	-	Bellis et al. [30]
C. (A.) brevitarsis	Solomon Islands	ww06180	KJ162971	-	-	-	-	-	Bellis et al. [30]
C. (A.) brevitarsis	China	ww06256	KJ162972	-	-	-	-	-	Bellis et al. [30]
C. (A.) brevitarsis	Australia	ww06046	KJ162974	-	-	-	-	-	Bellis et al. [30]
C. (A.) brevitarsis	East Timor	ww05959	KJ162975	-	-	-	-	-	Bellis et al. [30]
C. (A.) candolfii	Gabon	GALK10	KC986403	-	-	-	-	-	Delécolle et al. [69]
C. (A.) candolfii	Gabon	GALK11	KC986404	-	-	-	-	-	Delécolle et al. [69]
C. (A.) gulbenkiani	South Africa	ww14326	KJ162978	-	-	-	-	-	Bellis et al. [30]
C. (A.) hui	Papua New Guinea	ww15087	KJ162979	-	-	-	-	-	Bellis et al. [30]
C. (T.) humeralis	Japan	-	AB360993	AB360993	-	AB462282	-	-	Matsumoto et al. [37, 41]
C. (A.) imicola	China	ww24060	KJ162984	-	-	-	-	-	Bellis et al. [30]
C. (A.) jacobsoni	-	-	AB360991	AB360991	-	-	-	-	Matsumoto et al. [37]
C. (A.) jacobsoni	Papua New Guinea	ww12137	KJ162985	-	-	-	-	-	Bellis et al. [30]
C. (A.) kwagga	-	-	AF069237	-	-	-	-	-	Linton et al. [13]
C. (A.) kwagga	South Africa	ww14310	KJ162986	-	-	-	-	-	Bellis et al. [30]
C. (A.) kwagga	South Africa	ww14309	KJ162987	-	-	-	-	-	Bellis et al. [30]
C. (A.) loxodontis	-	-	AF069235	-	-	-	-	-	Linton et al. [13]
C. (A.) loxodontis	-	-	AF069236	-	-	-	-	-	Linton et al. [13]
C. (A.) loxodontis	South Africa	ww14312	KJ162988	-	-	-	-	-	Bellis et al. [30]
C. (A.) nudipalpis	East Timor	ww06172	KJ162989	-	-	-	-	-	Bellis et al. [30]

C. (A.) nudipalpis	East Timor	ww05928	KJ162990	-	-	-	-	-	Bellis et al. [30]
C. (A.) nudipalpis	East Timor	ww06168	KJ162991	-	-	-	-	-	Bellis et al. [30]
C. (A.) nudipalpis	East Timor	ww06164	KJ162992	-	-	-	-	-	Bellis et al. [30]
C. (A.) nudipalpis	East Timor	ww06155	KJ162993	-	-	-	-	-	Bellis et al. [30]
C. (A.) nudipalpis	East Timor	ww06158	KJ162994	-	-	-	-	-	Bellis et al. [30]
C. (A.) nudipalpis	East Timor	ww06157	KJ162995	-	-	-	-	-	Bellis et al. [30]
C. (A.) orientalis	East Timor	ww06223	KJ162997	-	-	-	-	-	Bellis et al. [30]
C. (A.) tainanus	-	-	AB360987	AB360987	-	AB462263	-	-	Matsumoto et al. [37, 41]
C. (T.) matsuzawai	-	-	AB364649	AB364649	-	AB462283	-	-	Matsumoto et al. [37, 41]
C. (A.) pseudopallidipennis	-	-	AY286329	-	-	-	-	-	Meiswinkel & Linton [25]
C. (A.) tuttifrutti	South Africa	-	AF069244	-	-	-	-	-	Linton et al. [13]
C. (A.) tuttifrutti	South Africa	-	AF069245	-	-	-	-	-	Linton et al. [13]
C. (A.) tuttifrutti	South Africa	ww14322	KJ162998	-	-	-	-	-	Bellis et al. [30]
C. (A.) tuttifrutti	South Africa	ww14321	KJ162999	-	-	-	-	-	Bellis et al. [30]
C. (A.) wadai	-	-	AB360997	AB360997	-	-	-	-	Matsumoto et al. [37]
C. (A.) wadai	Japan	ww09015	KJ163000	-	-	-	-	-	Bellis et al. [30]

Additional references:

68. Li GQ, Hu YL, Kanu S, Zhu XQ. PCR amplification and sequencing of ITS1 rDNA of *Culicoides arakawae*. Vet Parasitol. 2003;112:101-108.

69. Delécolle J-C, Paupy C, Rahola N, Mathieu B. Description morphologique et moléculaire d'une nouvelle espèce de *Culicoides (Avaritia)* du Gabon (Diptera, Ceratopogonidae) [in French]. Bull Société Entomol Fr. 2013;118:513–9.
